# Helicobacter heilmannii Infection With Concurrent Gastric Cancer: A Case Report

**DOI:** 10.7759/cureus.79729

**Published:** 2025-02-26

**Authors:** Tomohiro Kamio, Yoshiyasu Kono, Masaya Iwamuro, Tomoki Yoshikawa

**Affiliations:** 1 Department of Gastroenterology and Hepatology, Faculty of Medicine, Dentistry, and Pharmaceutical Sciences, Okayama University, Okayama, JPN; 2 Department of Gastroenterology and Hepatology, Okayama University Hospital, Okayama, JPN

**Keywords:** eradication, gastric cancer, gastritis, helicobacter heilmannii, non-helicobacter pylori helicobacter

## Abstract

*Helicobacter heilmannii *(*H. heilmannii*), a zoonotic pathogen, is increasingly recognized as a cause of gastritis and a potential risk factor for gastric cancer, despite its rarity. Here, we report a case of multiple synchronous early gastric cancers in a female patient in her 40s with *H. heilmannii*-associated gastritis. She underwent an esophagogastroduodenoscopy (EGD) for the evaluation of gastric discomfort. Endoscopy revealed cobblestone-like gastritis. Histological examination confirmed a signet ring cell carcinoma. *Helicobacter pylori *(*H. pylori*) antibody test was negative, and spiral-shaped bacteria were detected by Giemsa staining and real-time polymerase chain reaction (PCR), confirming *H. heilmannii* infection. Endoscopic submucosal dissection achieved curative resection without recurrence. *Helicobacter heilmannii*-induced gastritis presents distinct features, including a cobblestone-like appearance and lymphocyte-dominant infiltration, differing from *H. pylori*-associated gastritis. Chronic inflammation and immune modulation caused by *H. heilmannii* infection may contribute to carcinogenesis. Considering the diagnostic challenges and zoonotic transmission risks, enhanced awareness of *H. heilmannii*-associated gastric cancer is essential. This case highlights the importance of identifying *H. heilmannii *in *H. pylori*-negative gastritis and its potential role in gastric carcinogenesis. Further research is required to elucidate the pathogenic mechanisms and establish effective management protocols.

## Introduction

*Helicobacter pylori* (*H. pylori*) is well known for its role in chronic gastritis, peptic ulcers, and gastric cancer, and its eradication is the recommended strategy for gastric cancer prevention. In recent years, however, other species of *Helicobacter *that are not *H. pylori*, are collectively known as non-*H. pylori Helicobacter *(NHPH), have also been implicated in gastric disease [1-3]. Among these, *Helicobacter heilmannii *(*H.heilmannii*) is a zoonotic pathogen found in animals such as dogs, cats, and pigs. Human infection is rare, accounting for an estimated 0.1% to 0.6% of *Helicobacter *infections in developed countries; however, it is increasingly associated with chronic gastritis and may play a role in gastric carcinogenesis [4, 5].

*Helicobacter heilmannii*-associated gastritis presents distinct endoscopic and histological features compared with *H. pylori*, including a cobblestone-like appearance in the gastric antrum and lymphocyte-dominant infiltration with less neutrophilic activity. These unique features can aid in diagnosis, as conventional *H. pylori* tests such as rapid urease testing are typically negative in NHPH cases. Although the mechanisms linking *H. heilmannii *to gastric cancer are not fully understood, cobblestone-like gastritis has been suggested as a potential risk factor, particularly for undifferentiated carcinomas [6].

Here, we present a rare case of multiple synchronous early gastric cancers in a patient with *H. heilmannii*-associated gastritis, highlighting the importance of recognizing NHPH infections in gastric cancer risk assessment, even in *H. pylori*-negative patients. This report aimed to add to our understanding of the potential role of *H. heilmannii* in gastric carcinogenesis.

## Case presentation

The patient was a female in her 40s. She had a medical history of total hysterectomy for cervical cancer. The patient had no family history of any cancer. She had never smoked and occasionally consumed alcohol. She had been raising dogs and cats for several decades. She complained of epigastric discomfort, and esophagogastroduodenoscopy (EGD) was performed to investigate the cause of the symptom. A flat, pale lesion measuring 5 mm was identified at the anterior wall of the greater curvature of the gastric angle. The histological diagnosis based on forceps biopsy was a signet ring cell carcinoma. The patient was referred to our hospital for further evaluation and treatment.

No unusual physical or laboratory findings were observed. Serum *H. pylori* antibody test results were negative, and tumor markers (CEA, CA-19-9) were within the normal range (Table 1). A contrast-enhanced CT scan revealed no obvious metastasis.

**Table 1:** The patient's aboratory findings WBC: white blood cells; RBC: red blood cells; Hb: hemoglobin; PLT: platelet; PT: prothrombin time. PT-INR: prothrombin time-international normalized ratio; APTT: activated partial thromboplastin time; TP: total protein; ALB: albumin; AST: aspartate transaminase; ALT: alanine transaminase; ALP: alkaline phosphatase; LDH: lactate dehydrogenase; Na: sodium; K: potassium; Cl: chlorine; BUN: blood urea nitrogen; Cr: creatinine; CRP: C-reactive protein; CEA: carcinoembryonic antigen; CA 19-9: carbohydrate antigen-19; HP: *Helicobacter pylori*

Test	Observed value	Reference range
WBC	5.1×10^3^/μL	3.3-8.6×10^3^/μL
RBC	4.02×10^6^/μL	3.86-4.92×10^6^/μL
Hb	13.9 g/dL	11.5-14.8 g/dL
PLT	294×10^3^/μL	158-348×10^3^/μL
PT	102 %	73-118 %
PT-INR	0.99	
APTT	26.3 seconds	26.9-38.1 seconds
TP	7.0 g/dL	6.6-8.1 g/dL
ALB	4.4 g/dL	4.1-5.1 g/dL
T.Bil	0.5 mg/dL	0.4-1.5 mg/dL
AST	13 U/L	13-30 U/L
ALT	9 U/L	7-23 U/L
ALP	41 U/L	38-113 U/L
LDH	176 U/L	124-222 U/L
Na	142 mmol/L	138-145 mmol/L
K	3.5 mmol/L	3.6-4.5 mmol/L
Cl	106 mmol/L	101-108 mmol/L
BUN	10.8 mg/dL	8.0-20.0 mg/dL
Cr	0.58 mg/dL	0.46-0.79 mg/dL
CRP	0.02 mg/dL	< 0.15 mg/dL
CEA	2.08 ng/mL	< 5.0 ng/mL
CA 19-9	31.9 U/mL	0.0-35.4 U/mL
Serum anti-HP antibody titer	3 U/mL	< 10 U/mL

**Figure 1:**
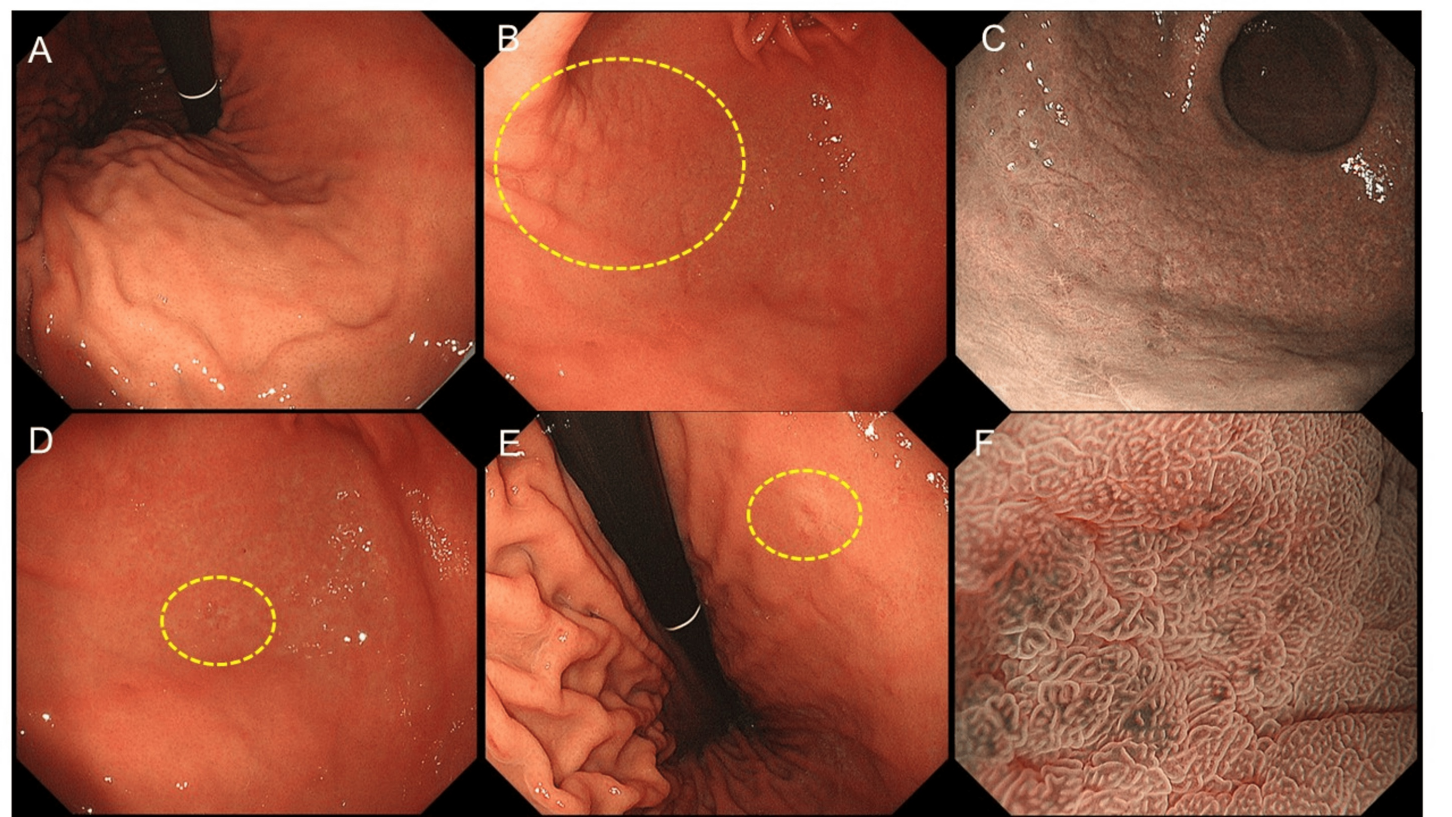
The patient's endoscopic images The scope used for the examination was the GIF-H290Z (Olympus Corporation, Tokyo, Japan) (A) Endoscopic image showing the regular arrangement of collecting venules in the gastric body without atrophic changes, typical in non-*Helicobacter pylori Helicobacter* infections. (B) White-light image and (C) narrow-band image showing a cobblestone-like appearance of the gastric antrum, with small elevated lesions and a fissured mucosal pattern, suggesting chronic gastritis due to *Helicobacter heilmannii* infection. (D) Endoscopic image showing the lesion previously diagnosed as a signet ring cell carcinoma at another hospital. (E) Endoscopic image showing the second flat, pale lesion (5 mm in diameter) located at the posterior wall of the lesser curvature of the gastric angle. This lesion was also diagnosed as a signet ring cell carcinoma. (F) Magnified image-enhanced endoscopic images of the lesion using narrow-band imaging.

The EGD was performed again at our hospital and showed the presence of a regular arrangement of collecting venules up to the lesser curvature of the gastric angle, with no atrophic changes in the gastric body mucosa (Figure 1A). Fissure-like mucosa was observed in the gastric antrum, and small elevations were prominent at the anterior wall of the greater curvature of the gastric antrum, showing a cobblestone-like appearance of gastritis (Figures 1B-1C). In addition to the lesion detected at the previous hospital (Figure 1D), another flat, pale-colored lesion, 5 mm in diameter, was found at the posterior wall of the lesser curvature of the gastric angle (Figure 1E). The magnified narrow-band image of the cancerous lesion is shown in Figure 1F. Histological diagnosis using forceps biopsy revealed signet ring cell carcinoma.

**Figure 2:**
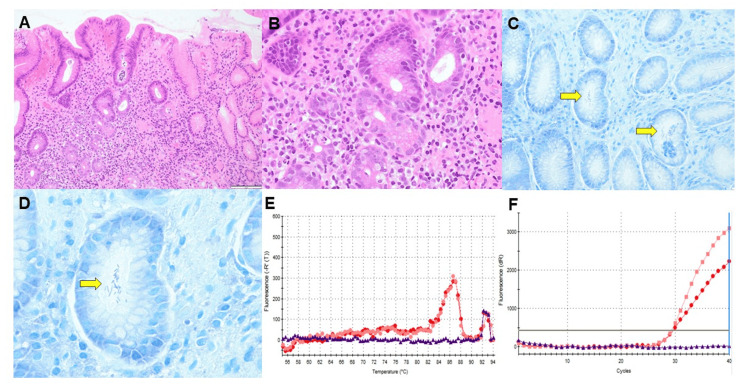
Histological images and polymerase chain reaction (PCR) amplification curves (A) and (B): Histopathological images of the non-cancerous gastric antrum showing mild atrophic changes according to the updated Sydney classification system, with mild inflammatory infiltration compared with typical Helicobacter pylori-associated gastritis. (C) and (D): Giemsa-stained sections revealing large spiral-shaped bacteria within the pyloric gland area, confirming *Helicobacter heilmannii* (*H. heilmannii*) infection. (E) and (F) Real-time PCR results confirming the presence of *H. heilmannii *DNA in the gastric biopsy specimen, establishing a definitive diagnosis of *H. heilmannii* infection.

A biopsy of the non-neoplastic area of the gastric antrum showed mild atrophic changes in the gastric mucosa according to the updated Sydney system [7] (evaluation of the antrum only), with mild infiltration of inflammatory cells compared with *H. pylori*-infected gastritis (Figures 2A-2B). Giemsa staining revealed large spiral bacteria in the pyloric gland area (Figures 2C-2D), suggesting NHPH infection, which was confirmed by real-time polymerase chain reaction (PCR) as *H. heilmannii* (Figures 2E-2F). Based on these findings, the patient was diagnosed with early gastric cancer that developed concurrently with cobblestone gastritis caused by *H. heilmannii* infection. 

**Figure 3:**
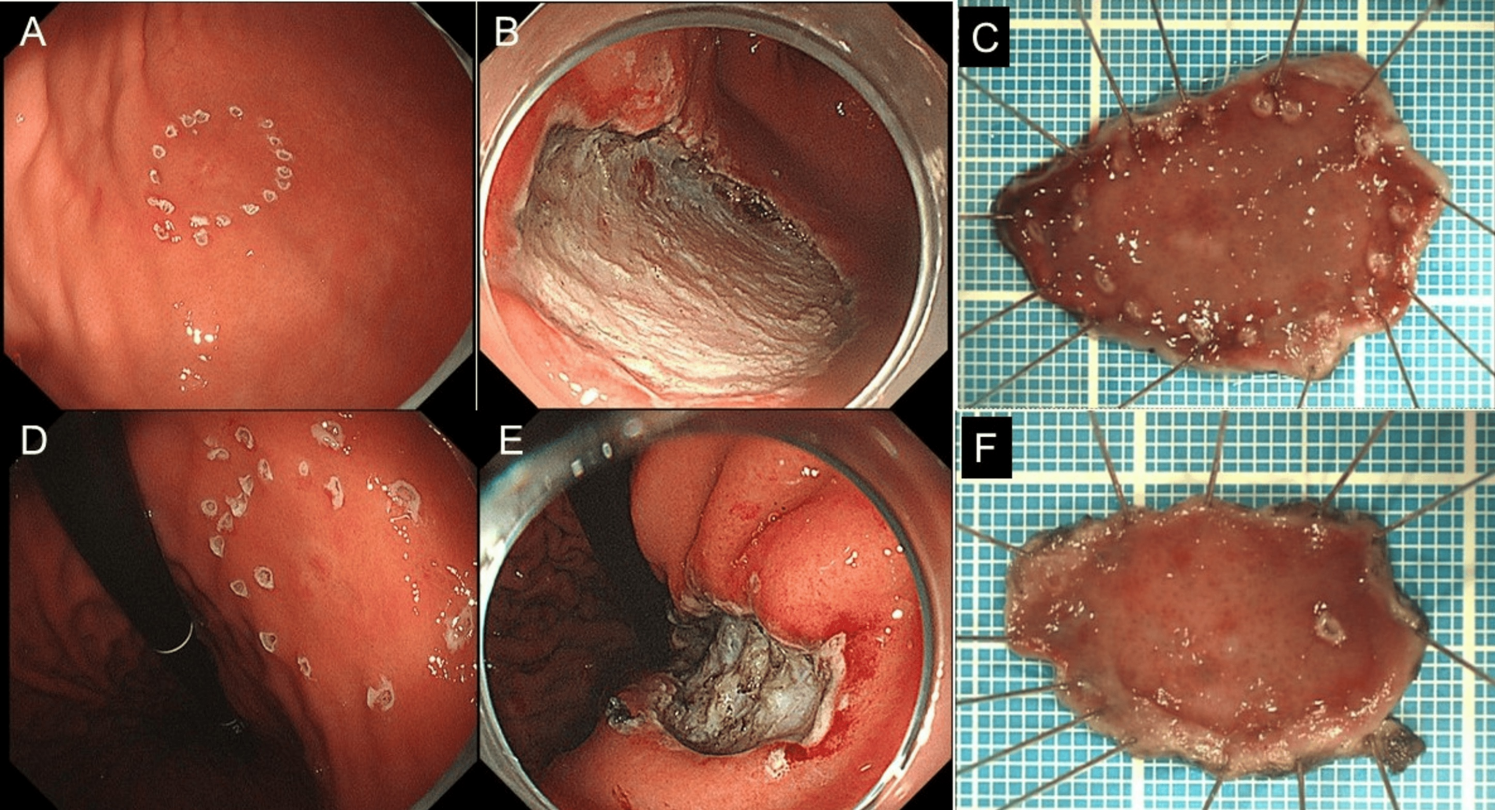
Endoscopic images during gastric endoscopic submucosal dissection and photographs of the resected specimen (A) Endoscopic image after marking of the first lesion; (B) Endoscopic image of the ulcer after resection of the first lesion; (C) Image of the resected specimen of the first lesion; (D) Endoscopic image after marking of the second lesion; (E) Endoscopic image of the ulcer after resection of the second lesion; (F) Image of the resected specimen of the second lesion

The patient underwent endoscopic submucosal dissection for the two lesions (Figure 3). The pathological findings were pT1a, pUL0, Ly0, V0, pHM0, pVM0, and pStage IA in both lesions, resulting in successful curative resection.

## Discussion

Non-*H. pylori Helicobacter *infections are uncommon in humans, with *H. heilmannii* being a notable example associated with gastric diseases [1-3]. Unlike *H. pylori*, which is well established as a causative factor for various gastric conditions, the role of NHPH, particularly *H. heilmannii*, in gastric carcinogenesis remains unclear owing to its rarity and difficulty in detection. This case of multiple synchronous early gastric cancers occurring in *H. heilmannii*-infected gastritis provides insights into the potential association between NHPH and gastric cancer.

*Helicobacter heilmannii i*s known to naturally infect animals, especially dogs, cats, and pigs. Human infections likely result from close contact with these animals, which is consistent with our patient's long-term pet ownership [4, 5]. Endoscopically,* H. heilmannii* infection often presents distinct characteristics from *H. pylori* infection, including the absence of atrophy in the gastric body and a unique cobblestone-like appearance or fissured mucosa in the antrum [8-11]. These features were evident in the present case, in which cobblestone gastritis was observed along with two early-stage carcinomas in the gastric angle. This has been postulated to arise from immune responses specifically induced by *H. heilmannii*, leading to inflammation and altered gastric mucosal architecture [6].

Although the exact mechanisms underlying *H. heilmannii*-induced gastric cancer remain unknown, several hypotheses have been proposed. First, chronic inflammation and immune modulation due to persistent bacterial infections may contribute to an environment conducive to carcinogenesis. *Helicobacter heilmannii *infection often results in lymphocyte-dominant infiltration, a pattern that is distinct from the neutrophil-rich response typically observed in *H. pylori* infection [12, 13]. This type of inflammation can predispose patients to carcinogenesis, particularly in the presence of cobblestone-like gastritis, which is associated with undifferentiated gastric cancer. Second, co-infection with *H. pylori* has been suggested as a potential factor in some cases, although this was not observed in our patient. Studies have indicated that the co-existence of multiple *Helicobacter *species increases the risk of malignancy, possibly through synergistic inflammatory effects or enhanced mucosal damage [14]. However, given the negative *H. pylori *antibody results in this patient, it is more likely that carcinogenesis was directly associated with *H. heilmannii* infection.

Histopathologically, *H. heilmannii*-infected gastritis tends to display milder inflammatory cell infiltration with larger, spiral-shaped bacteria that can be identified using special stains such as Giemsa. In contrast to *H. pylori*-related gastritis, lymphocytic predominance in the inflammatory infiltrate has been noted. This distinct pattern is valuable in differential diagnosis, particularly in cases where typical *H. pylori*-related features are absent but the risk of gastric cancer remains high. Additionally, genetic predispositions such as hereditary diffuse gastric cancer were considered; however, no family history or clinical findings supporting a genetic cancer predisposition were identified.

Diagnosing *H. heilmannii* infection is challenging because of the limited sensitivity of conventional tests, such as rapid urease and urea breath tests, which are optimized for *H. pylori *detection. Although useful, real-time PCR and culture methods are not routinely used in clinical practice [15, 16]. Thus, there is a need for enhanced awareness of the signs of NHPH infection among clinicians, especially given the unique endoscopic features of *H. heilmannii.*

Therapeutically, *H. heilmannii *eradication regimens are generally based on *H. pylori *protocols. However, the effectiveness and long-term impact of eradication on disease prevention remain unclear [17]. Given the zoonotic nature of *H. heilmannii*, re-infection may occur if there is environmental exposure, such as close contact with animals. Therefore, eradication treatment considerations should include patient lifestyle and potential re-exposure risks.

## Conclusions

In conclusion, this case emphasizes the importance of considering NHPH infection, particularly* H. heilmannii*, in cases of gastric cancer with non-*H. pylori*-related gastritis features. The appearance of cobblestone-like gastritis combined with chronic inflammation may contribute to the development of gastric cancer, underscoring the need for vigilance in diagnosing and managing NHPH infections. Further studies are necessary to clarify the oncogenic potential of* H. heilmannii* and to establish effective diagnostic and therapeutic protocols for NHPH-associated gastric diseases.
